# Numerical Investigation on Head and Brain Injuries Caused by Windshield Impact on Riders Using Electric Self-Balancing Scooters

**DOI:** 10.1155/2018/5738090

**Published:** 2018-03-25

**Authors:** Shi Shang, Yanting Zheng, Ming Shen, Xianfeng Yang, Jun Xu

**Affiliations:** ^1^Advanced Vehicle Research Center (AVRC), Beihang University, Beijing 100191, China; ^2^Department of Automotive Engineering, School of Transportation Science and Engineering, Beihang University, Beijing 100191, China; ^3^Department of Transportation, School of Transportation Science and Engineering, Beihang University, Beijing 100191, China; ^4^Bioengineering Center, Wayne State University, Detroit, MI 48201, USA; ^5^Institute of Solid Mechanics, Beihang University, Beijing 100191, China

## Abstract

To investigate head-brain injuries caused by windshield impact on riders using electric self-balancing scooters (ESS). Numerical vehicle ESS crash scenarios are constructed by combining the finite element (FE) vehicle model and multibody scooter/rider models. Impact kinematic postures of the head-windshield contact under various impact conditions are captured. Then, the processes during head-windshield contact are reconstructed using validated FE head/laminated windshield models to assess the severity of brain injury caused by the head-windshield contact. Governing factors, such as vehicle speed, ESS speed, and the initial orientation of ESS rider, have nontrivial influences over the severity of a rider's brain injuries. Results also show positive correlations between vehicle speed and head-windshield impact speeds (linear and angular). Meanwhile, the time of head-windshield contact happens earlier when the vehicle speed is faster. According to the intensive study, windshield-head contact speed (linear and angular), impact location on the windshield, and head collision area are found to be direct factors on ESS riders' brain injuries during an impact. The von Mises stress and shear stress rise when relative contact speed of head-windshield increases. Brain injury indices vary widely when the head impacting the windshield from center to the edge or impacting with different areas.

## 1. Introduction

The electric self-balancing scooter (ESS) has been attracting much attention because of its convenience and the increasing demand for modern portable transportation tools. The safety performance of ESS during traffic accidents has also been investigated because ESS riders have been considered as one group of vulnerable road users (VRUs). ESS riders may suffer from severe injuries during vehicle ESS accidents [1].

Scientists and engineers have continuously paid much attention to pedestrians/cyclists' head-brain injuries to investigate the impact mechanism and reduce casualties by designing pedestrian-friendly automobiles [2–4]. However, only a few studies on ESS safety have been conducted. Xu et al. [5, 6] first analyzed the ESS riders' head injuries caused by vehicular or ground impact. Under the same impact situation, the ESS rider's head impacts the windshield 20~60 ms later compared to the pedestrian.

The windshield contributes the highest frequency (32%) of the head crash zone in vehicle-pedestrian accidents [7]. Previous studies intensively investigated the characteristics of head-windshield contacts [8, 9], such as head form-windshield impacts tests [10–12], responses of windshield [3, 13, 14] (Alvarez and Kleiven [3] compared two kinds of windshield modelling approaches to capture the head form accelerations and windshield deformations from head impacts and found that simple plasticity models of the windshield are not sufficient to predict that a nonlocal failure model [15] was needed), head-windshield contact reconstructions, and head injury analysis using FE methods [9, 16] (Mordaka et al. [17] analyzed three cases of pedestrian head-to-windshield impact accidents and performed additional parametric studies using a detailed FE model of the head). Results have confirmed that the severity of pedestrians/cyclists' head injuries is influenced by numerous factors involving vehicle speed [2, 18] and vehicle type [19]. However, very few comparative studies about the ESS riders' head injuries caused by vehicles could be found. A preliminary study has been conducted in which the head injury of an ESS rider caused by windshield impact was examined [6]. The aim of the study is to evaluate the safety of electric self-balancing scooters (ESSs) through examining head-brain injuries caused by vehicle contact and understanding how the factors influence the effect on the severity of the ESS rider's brain injury.

## 2. Materials and Methods

### 2.1. Accident Scenario Setup

A two-phase simulation methodology is adopted to evaluate the ESS riders' brain injury caused by vehicle impact.

**Figure 1:**
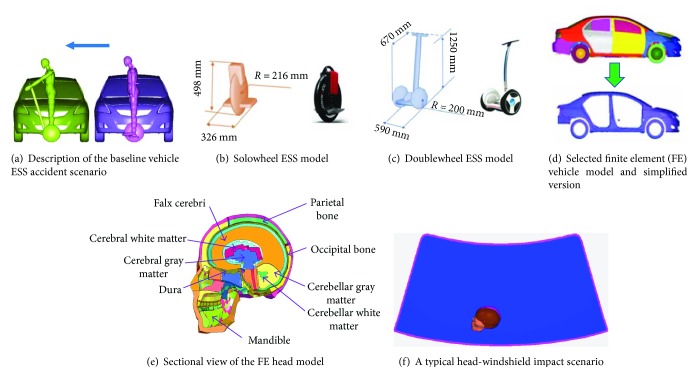
Description of numerical models.

First, vehicle ESS rider impacts accident scenes are first numerically reconstructed are set up on MADYMO [20] platform (version 7.5), which is most commonly used in vehicle safety [9, 21, 22]. This multirigid body simulation basically provides the kinematics of the ESS rider which serves as the boundary conditions for the subsequent FE simulation. The lateral impact is set as the baseline vehicle ESS collision accident scenario, because this case accounts for the largest portion of vehicle VRU crashes in real-world accidents [9, 23]. The facing direction of the ESS rider is angled at 90° to the direction of vehicle movement (Figure 1(a)). A continuous brake with 0.8*g* deceleration, which assumed good contact friction between the tire and pavement [24], is adopted in all numerical models. The head-brain injuries of riders are examined comprehensively by varying the vehicle impact speed and ESS moving speed to represent diverse impact conditions.

Then, the biomechanical responses of the brain caused by head-windshield contact in vehicle ESS crash accident are examined. The processes of head-windshield contact are reconstructed and simulated using validated FE head [25] and windshield models [26] using LS-DYNA (version 971 R6.1.0). Similar simulation strategy was adopted in previous studies [9].

### 2.2. ESS Models

Two representative types of ESSs, namely, solowheel and doublewheel scooters, are selected and modeled as target scooters in the MADYMO platform. The multibody solowheel ESS model has one rigid body with three ellipsoids to depict its outer profile (Figure 1(b)), while the doublewheel ESS model has six ellipsoids to represent its profile (Figure 1(c)). A summary of the material stiffness values of the doublewheel and solowheel ESSs is listed in Tables 1 and 2, respectively. The friction coefficient between the ESS model and the pedestrian model was set as 0.3. The friction coefficient for the ESS wheels against the ground is also set as 0.3. The models, along with the parameters, have been applied in previous studies [5, 6].

### 2.3. Human Model

The 50th percentile male pedestrian model of MADYMO human database [27] is chosen as the ESS rider human model for the process of vehicle ESS contact. The validated pedestrian model [28] is widely used in vehicle VRU accident simulations and analysis [6, 22] to predict the injury and capture the kinematic response. Both solowheel and doublewheel ESS riders were set as standing posture, as shown in Figure 1(a). The human model manual [27] may be used to obtain more detailed information on anthropometry, configuration and contact, and so on.

### 2.4. Vehicle Model

One of the most popular vehicles, sedan, is selected as the model car in the vehicle impact simulations. The FE model of the sedan was developed by the National Crash Analysis Center of George Washington University under a contract with the FHWA and NHTSA of the US DOT [29]. Only the outer surface of the vehicle front-end is needed for vehicle ESS rider contact, and the weight of the ESS with the human is trivial compared to that of the vehicle. Thus, the FE vehicle model is simplified (Figure 1(d)) to retain the outer profile to considerably save computation time [6]. In the MADYMO platform, the outer surface was employed as the rigid finite element and the original contact stiffness was kept. The contact types between the vehicle, ESS, and human are all set as a combined contact. Slave contact is applied for the type of human-ground contact and vehicle wheel-ground contact.

### 2.5. FE Head-Windshield Impact Model

The processes of head-windshield contacts are reconstructed using validated FE windshield and human head model to extensively evaluate the head-brain injury of an ESS rider. This method has been frequently used in vehicle-pedestrian crash accident reconstructions [9, 30]. The THUMS adult male 50th percentile pedestrian head is picked as the head model. The THUMS pedestrian model version 4.0 (the head model followed THUMS version 3.0 developed and validated by Kimpara et al. [25]) is developed by the Toyota Motor Corporation and Toyota Central R&D Labs [31] based on real anthropometric parameters and cadaveric tests which may well represent an actual pedestrian involved in a traffic accident to investigate the safety problems. The head model contains skull, skin, scalp, cerebrum, cerebellum, mandible, teeth, meninges, and so on The cross-section of the THUMS head model in the median sagittal plane is shown in Figure 1(e).

The FE windshield model in this paper is adopted from Xu et al. and Yu et al. [26, 32]. The model has three layers, that is, the polyvinyl butyral (PVB) interlayer is sandwiched between two glass sheet layers. The thicknesses of the three layers from the outside to the inside faces of the windshield are 2.55 mm, 0.76 mm, and 2.10 mm, respectively. The glass model is modeled as a shell element, whereas the PVB interlayer is modeled as a solid element with a mesh size of 5 mm × 5 mm. A strain failure is added to the laminated windshield model (Table 3) to simulate a more realistic evolution of a cracked windshield during impact which may affect the head injury. The strain rate dependency of the PVB interlayer material is also considered in this model. Accordingly, MAT 123 “MAT_PIECEWISE_LINEAR_PLASTICITY,” a linear elastic model of LS-DYNA material database, is selected to characterize the material characteristics of PVB and glass during dynamic impact simulations. The parameter settings are summarized in Table 3. During the head-to-windshield impact, the lower glass bears the tensile stress and fails first, while the upper glass bears the compression force and fails accordingly. Thus, the mechanical properties of the upper and lower glass are comprehensively determined by an extensive numerical evaluation referring to all dynamic compression and tensile test curves at different strain rates [33]. The upper glass plastic failure strain (0.0004) and the lower glass plastic failure strain (0.00024) are determined by an extensive numerical assessment based on compressive and tensile experimental data at different strain rates, respectively. According to a previous windshield test and a simulation study, the glass plastic failure strain ranges from 0.0001 to 0.001 [33–37]. Thus, the difference of properties between the upper glass layer and lower glass layer in this study is reasonable. In addition, the boundary condition of the windshield is fully constrained, and the relative impact speed and position between the head and the vehicle of MADYMO output are employed as the input speed [17] (both linear and angular velocity with different *x*, *y*, and *z* components) of the FE head model in this paper. The contact type between the FE head and windshield models is set as “CONTACT_AUTOMATIC_SURFACE_TO_SURFACE” algorithm with a friction coefficient of 0.1 [26]. Yu et al. [32] have validated that the FE windshield models were devised to provide a highly realistic cracking morphology with an enhanced impact response for the laminated windshield compared to the experimental tests. In addition, the windshield deflection and head form impactor acceleration profiles are much more realistic than those obtained in previous studies.

### 2.6. Injury Evaluation Index

Cerebral contusion and laceration of the brain, that is, coup and contrecoup contusions, belonging to traumatic brain injury, can be the result of a direct impact to the head [38, 39]. Important brain parameters, such as coup pressure *P*_C_, contrecoup pressure *P*_CC_, von Mises stress *σ*_VM_, and maximum shear stress *τ*, obtained from FE simulation have strong correlations with the risk of AIS 3+ brain injuries [9]. The reference values of stresses or pressures in the current study are derived from Yao et al. [9], that is, *P*_C_, *P*_CC_, *σ*_VM_, and *τ* are 256, −152, 14.8, and 7.9 kPa, respectively.

## 3. Results and Discussion

**Figure 2:**
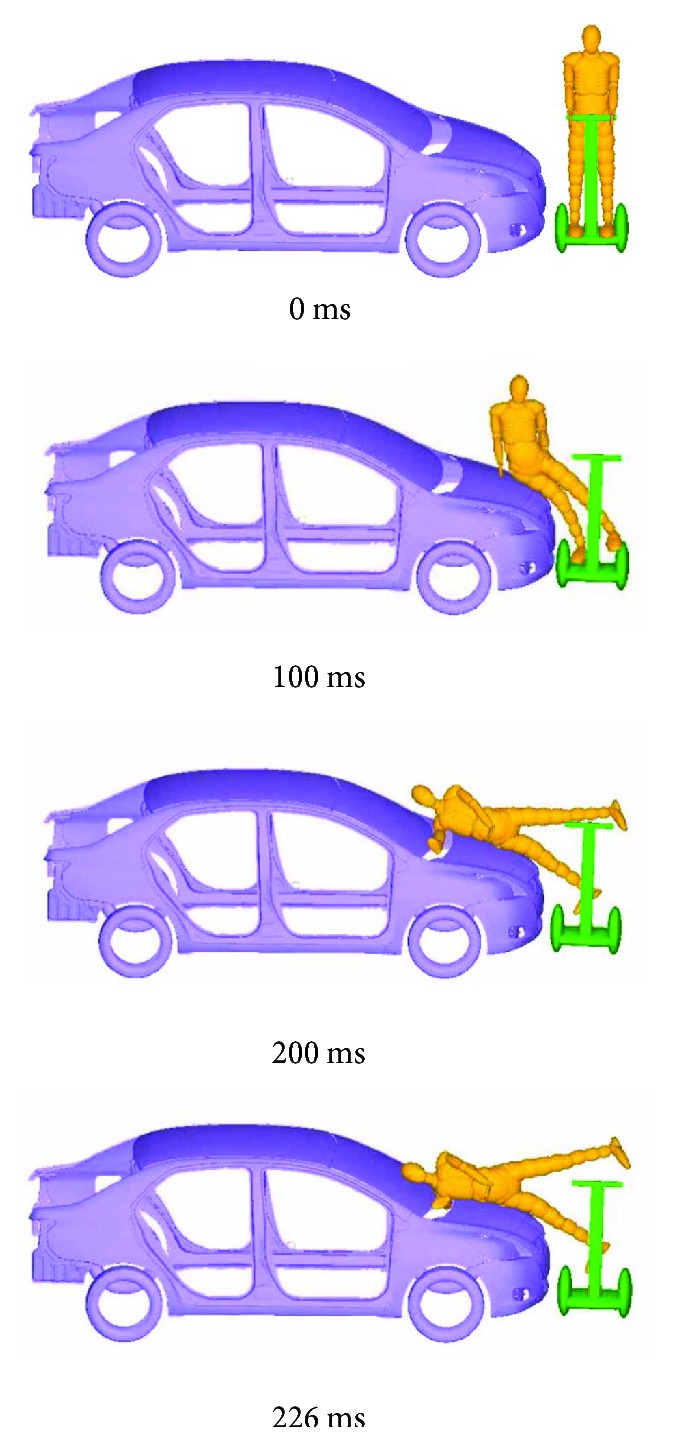
Vehicle-ESS impact processes under vehicle impact speed of 7 m/s.

**Figure 3:**
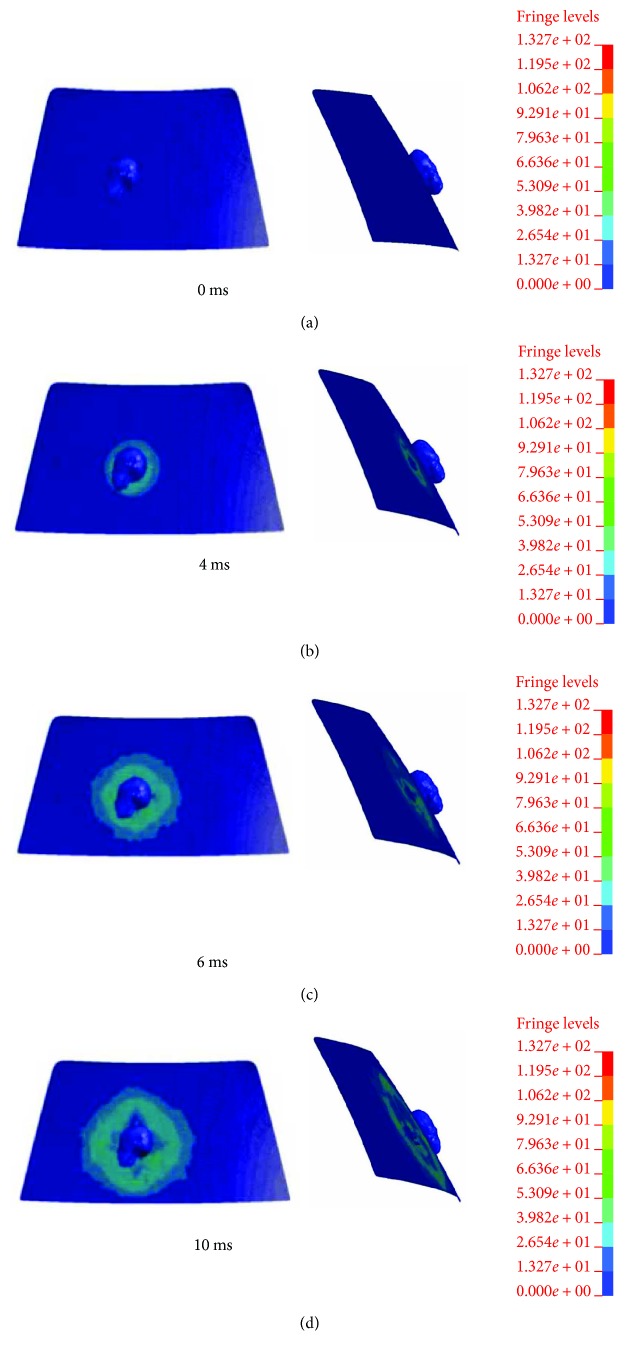
Variations in the von Mises stress of the windshield under vehicle impact speed of 7 m/s.

**Figure 4:**
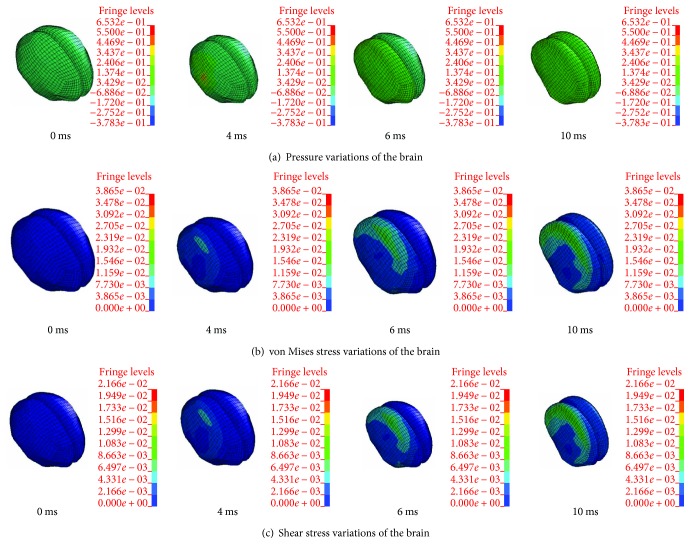
Variations in the pressure and stress of the doublewheel ESS rider's cerebrum under vehicle impact speed of 7 m/s.

Take one numerical simulation for example.  Figure 2 shows the impact process of vehicle doublewheel ESS accident under impact speed of *V*_C_ = 7 m/s. It can be observed that the ESS rider fell onto the bonnet after being hit by the vehicle, then, head-windshield contact occurred at 226 ms. The cerebrum of an ESS rider suffers sustained pressures and stresses during head-windshield impact.  Figure 3 shows the von Mises stress variations in the windshield. Figures 4(a)–4(c) show the pressure and stress variations in the doublewheel ESS rider's cerebrum.

The pressure quickly appears and diffuses in the collision half-side of the cerebrum when the initial head-windshield contact occurs. The cerebrum presents an obvious large deformation at 6 ms, and the pressure has diffused to the whole brain region. At the last stage demonstrated in Figure 4, the cerebrum pressure increases continuously, and the deformation of the cerebrum reaches an extremum. A concentration of pressure can be observed on the collision side of the cerebrum shortly after the stress wave transmitted from the scalp to the skull and further through CSF to the cerebrum. Then, the stress has diffused to almost half of the cerebrum at 6 ms. At the next stage, that is, at 10 ms, the equivalent effective stress has diffused to the other side and reaches a maximum value (38.6 kPa). The variation in the shear stress contour of the cerebrum is highly similar to the von Mises stress field distribution profile.

**Figure 5:**
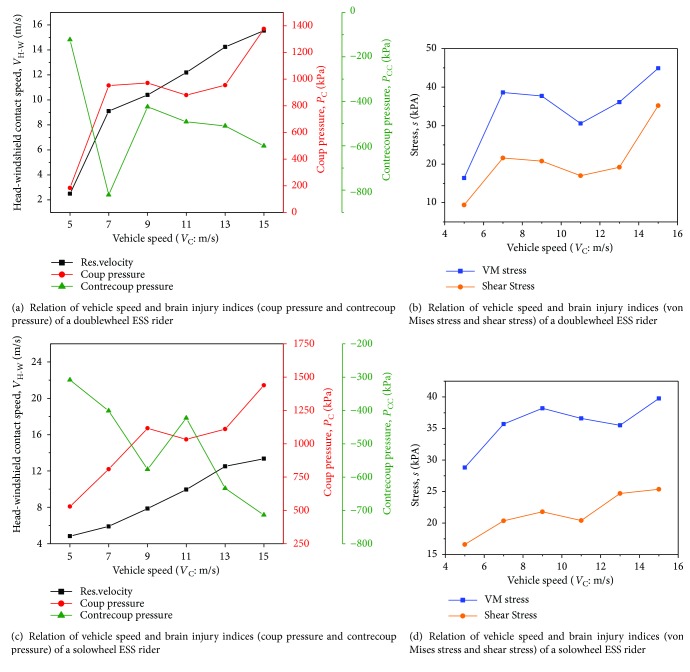
Relation of vehicle speed and brain injury index of ESS riders at side impact baseline cases.

Vehicle impact speed is widely accepted as the leading factor [5, 40] for the severity of VRU head injuries during vehicular accidents. Therefore, a study of vehicle speed effect on brain injury was carried out. In the setting of MADYMO simulations, baseline vehicle ESS accident scenario (see Figure 1(a)) was employed. Six vehicle speeds (*V*_C_ = 5, 7, 9, 11, 13, and 15 m/s) are considered to investigate the speed effect on the severity of ESS riders' head injuries caused by windshield collision. The boundary conditions of head-windshield impact were captured and accordingly employed to FE models. The relationships between *V*_C_ and *P*_C_, *P*_CC_, *σ*_VM_, and *τ* of ESS riders are illustrated in Figures 5(a) and 5(b), respectively.

**Figure 6:**
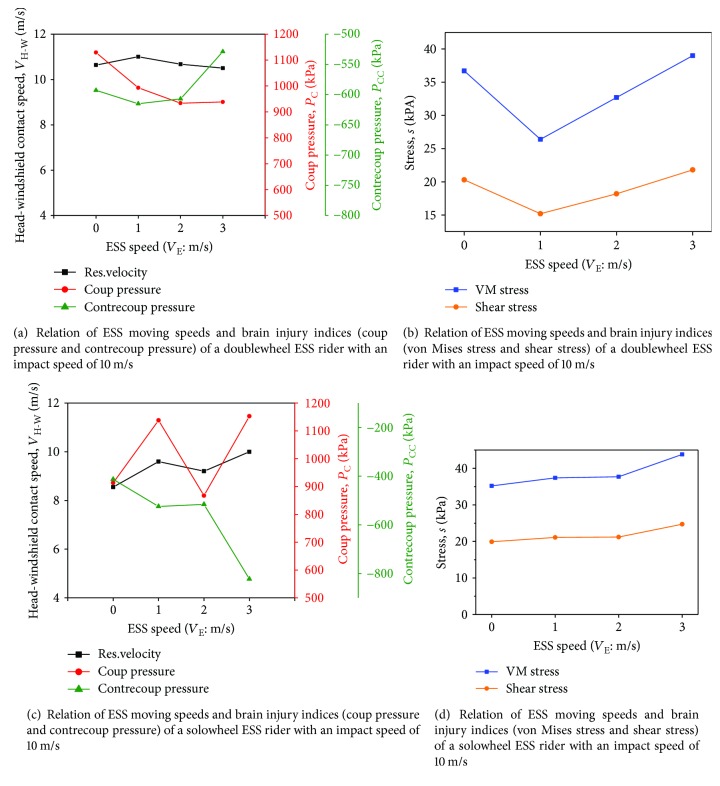
Relation of ESS moving speeds and brain injury indices of ESS riders under the impact speed of 10 m/s.

Changes in the ESS moving speed (*V*_E_) can lead to a change in the ESS riders' postimpact posture, thus affecting the severity of head-brain injuries. Similarly, a relative study was carried out to investigate ESS speed effect on brain injury. Vehicle ESS accident scenario was set as the baseline as shown in Figure 1(a). In addition, moving speeds were added in ESS and human models. Four ESS moving speeds (*V_E_* = 0, 1, 2, and 3 m/s) are chosen as the input parameters to investigate the effect of ESS moving speed on brain injury at *V*_C_ = 10 m/s during head-windshield impact. The relationships between *V*_E_ and *P*_C_, *P*_CC_, *σ*_VM_, and *τ* of doublewheel and solowheel ESS riders are presented in Figures 6(a) and 6(b), respectively.

### 3.1. Effect of Vehicle Speed on Brain Injury

**Figure 7:**
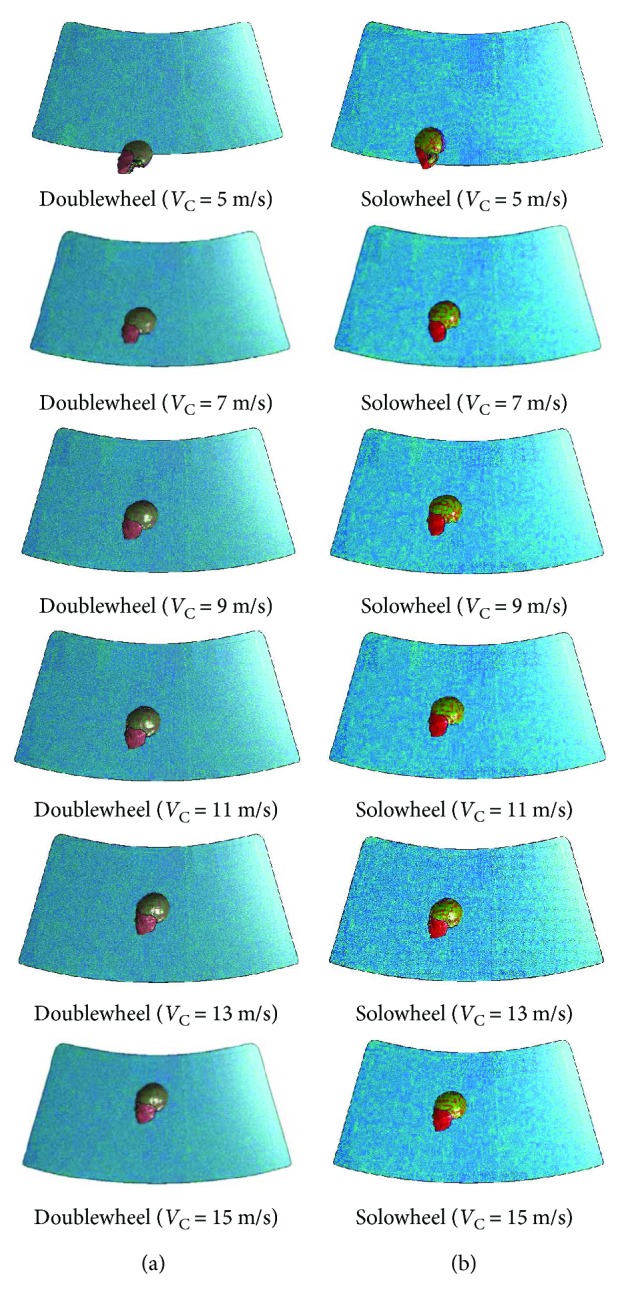
Head collision points on windshield under different vehicle impact speeds.

Figure 5 shows the relative speed between the head and the windshield, that is, *V*_H‐W_ increases with *V*_C_. In addition, *P*_C_, *P*_CC_, *σ*_VM_, and *τ* also generally have positive correlations with *V*_C_. As shown in Figures 5(a) and 5(b), there is a sharp increase of *V*_H-W_ from *V*_C_ = 5 m/s to 7 m/s, which could explain the high absolute value of contrecoup pressure as well as other brain injury indices. In addition, as LS-PrePost of FE simulations show, when the head impacts the windshield, the centralized pressure on the cerebrum moves relatively faster from the impact side to the offside in the two cases, resulting in the high absolute values of contrecoup pressure. By examining the MADYMO output animations and the collision times (Table 4) of head-windshield contacts, there is a big head-windshield impact time difference (71 ms) between solowheel cases with *V*_C_ = 5 m/s and 7 m/s, which makes a relatively large speed gap between the two vehicle-windshield contacts. The head-windshield contact points (as shown in Figure 7) may also change with the impact speed. For instance, the impact points are in the lower zone of the windshield when *V*_C_ = 5 m/s moves to a much higher position at *V*_C_ = 15 m/s. It indicates that *V*_H-W_ and head contact location on windshield influences the severity of brain injuries. Therefore, these two parameters were discussed in detail in the following sections of this study.

### 3.2. Effect of ESS Moving Speed on Brain Injury

**Figure 8:**
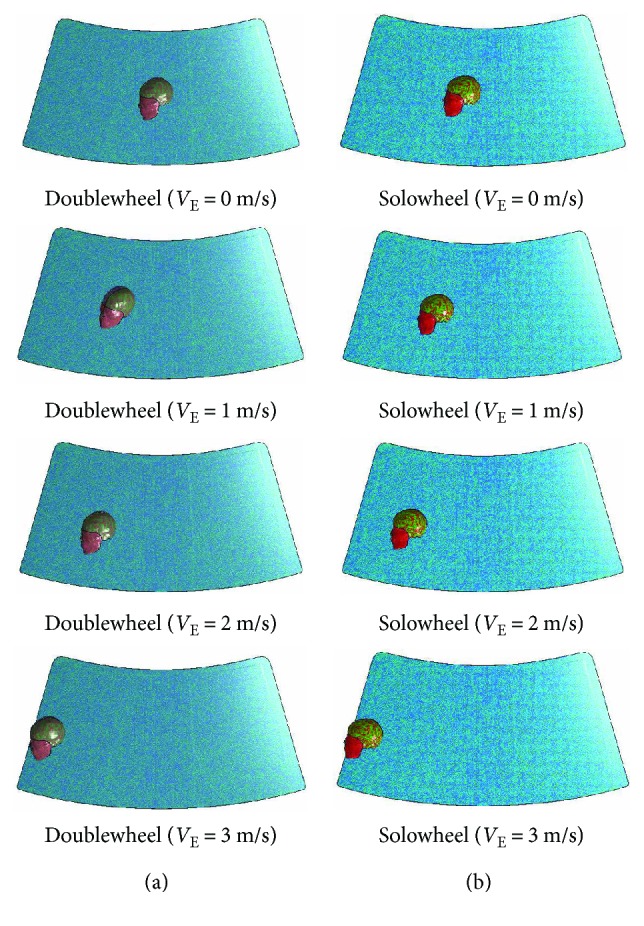
Head collision points on windshield under different ESS moving speeds.

As shown in Figure 6, brain injury indices are relatively lower at *V*_E_ = 0 m/s, and *P*_C_, *P*_CC_, *σ*_VM_, and *τ* show no obvious correlations with *V*_E_. However, *V*_E_ and brain injury indices are generally correlated in solowheel cases except the suddenly increased coup pressure when *V*_E_ = 1 m/s. The MADYMO output results show that locations of head and windshield impact are also strongly influenced by ESS moving speed. By examining the FE head-windshield impact simulation, when *V*_E_ = 1 m/s, the head has a lager deformation compared to the situation of ESS and the speed is zero, which may take the responsibility to the high value of coup pressure. Moreover, it can be speculated that with the increase of *V*_E_, *V*_H-W_ changes not much (within a fluctuation range of 2 m/s, as shown in Figure 6) while the impact locations on the windshield can vary widely (see Figure 8). It strengthens the importance to investigate the effect of the impact location of the windshield on the ESS rider's brain injury.

### 3.3. Effect of Vehicle ESS Impact Angle on Brain Injury

**Figure 9:**
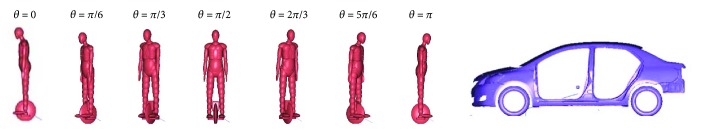
Description of different vehicle ESS impact angles under the impact speed of 10 m/s.

A previous study showed that the relative angle (*θ*) between the vehicle speed orientation and the ESS rider's facing direction has a major impact on the head contact regions and HIC_15_ values, as demonstrated in a previous study [5]. A series of parametric studies are performed to evaluate the influence of the impact angle *θ* when *V*_C_ = 10 m/s. The seven impact angles (*θ* = 0, *π*/6, *π*/3, *π*/2, 2*π*/3, 5*π*/6, and *π*) are illustrated in Figure 9.

**Figure 10:**
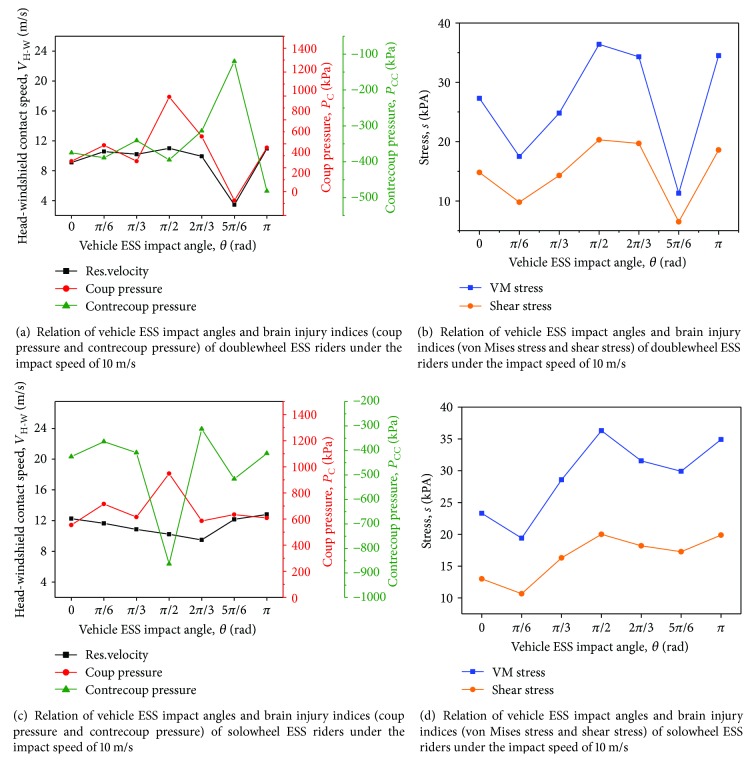
Relation of vehicle ESS impact angles and brain injury indices of ESS riders under the impact speed of 10 m/s.

The relations of *θ* and *P*_C_, *P*_CC_, *σ*_VM_, and *τ* are presented in Figure 10. *V*_H‐W_ does not remarkably vary in most doublewheel cases, except at *θ* = 5*π*/6 if the impact speed between the ESS rider and vehicle is constant. The variation in the brain injury indices is consistent with that of *V*_H‐W_. However, in solowheel cases, *V*_H‐W_ is more sensitive to the changes in *θ*, and no obvious correlation between *V*_H‐W_ and brain injury indices is found. The MADYMO output results show that the head contact location plays an important role in brain injury.

### 3.4. Effect of Head Impact Speed (Linear Velocity) on Brain Injury

**Figure 11:**
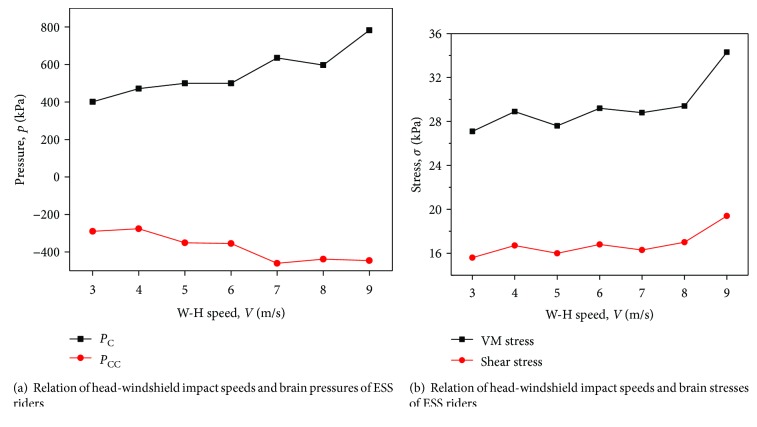
Relation of head-windshield impact speeds and brain injury indices of ESS riders under a typical impact scenario.

Previous studies showed that head impact speed is an important factor that influences VRU head-brain injuries during impact [41]. *V*_H‐W_ is highly dependent on the vehicle impact speed *V*_C_. Consequently, higher *V*_C_ produces more serious brain injuries caused by windshield contact. *V*_H‐W_ is first parametrically analyzed in this section. A typical impact scenario of an ESS rider's head-windshield contact is employed (shown in Figure 1(f)). *V*_H‐W_ varies from 3 m/s to 9 m/s with a vertical orientation.  Figure 11 shows the relationship between *V*_H‐W_ and *P*_C_, *P*_CC_, *σ*_VM_, and *τ*. The brain injury indices are positively correlated with *V*_H‐W_, indicating that the risk of suffering more serious brain injuries increases with impact speed.

### 3.5. Effect of Head Impact Speed (Angular Velocity) on Brain Injury

**Figure 12:**
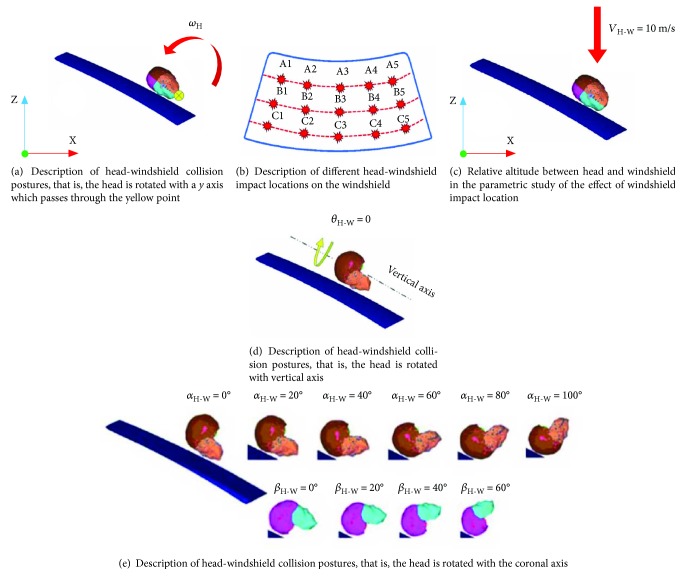
Description of different head-windshield impact situations.

**Figure 13:**
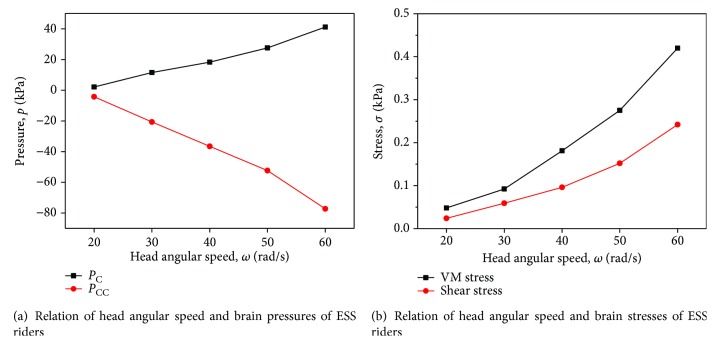
Relation of head angular velocities and brain injury indices of ESS riders under a typical impact scenario.

According to the MADYMO outputs, besides the linear velocity of the head, the angular velocity is also easy to be affected. Therefore, the effect of head impact angular velocity on brain injury is parametrically studied in this section. The impact scenario of head-windshield contact is in Figure 12(a) in which the angular velocity *ω*_H_ varies from 20 rad/s to 60 rad/s. The relationship between *ω*_H_ and brain injury indices is illustrated in Figure 13. It can be observed that the values of *P*_C_, *P*_CC_, *σ*_VM_, and *τ* are positively correlated *ω*_H_ but the influence is less compared with linear speed.

### 3.6. Effect of the Impact Location on the Windshield on Brain Injury

As mentioned in previous sections, further research of the impact location of windshield's effect on brain injury is warranted. Fifteen collision points on the outer surface of a windshield are evenly assigned (Figure 12(b)) to study the influence of these points on brain injury. In this parametric study, the orientation of *V*_H‐W_ is set as perpendicularly down with a speed of 10 m/s. Meanwhile, the relative altitude between the head and the windshield is illustrated in Figure 12(c). Brain injury indices obtained in each impact point case are listed in Table 5. The results show that *P*_C_, *P*_CC_, *σ*_VM_, and *τ* are relatively high when ESS riders' head impacts the areas of A1, B3, B5, and C5. The average values of *P*_C_, *P*_CC_, *σ*_VM_, and *τ* are 772.42 ± 93.4, −367 ± 232.54, 36.38 ± 1.27, and 19.82 ± 0.94 kPa, respectively. These values indicate that the collision region of the windshield has a great influence over *P*_CC_ but slightly influences *P*_C_, *σ*_VM_, and *τ*. Note that the surface of the windshield is convex rather than plane, changing the impact location on the windshield will alter the contact surface of the head model with the process of collision. In addition, boundary conditions of the windshield may also influence the biomechanical response of the brain.

### 3.7. Effect of Head Impact Region on Brain Injury

MADYMO output animations of a previous study (Section 3.3), which involves ESS rider's initial orientation effect on brain injury, showed the orientation of the head, that is, head impact region, which remarkably varies when the contact with windshield happened. In addition, the kinematic postures of ESS riders vary greatly with large uncertainties under diverse impact conditions [6]. Therefore, parametric studies are conducted to investigate the effect of head impact region on brain injury. The first case considered is when the ESS rider's head is facing toward the outer surface of the windshield, that is, *θ*_H‐W_ = 0 (Figure 12(d)). Other head-windshield collision postures (*θ*_H‐W_ = 0, *π*/6, *π*/3, *π*/2, 2*π*/3, 5*π*/6, and *π*) are adopted by rotating the FE head model around the vertical axis. A series of parametric studies are conducted based on different head impact regions at the same vertical head-windshield impact speed (*V*_H‐W_ = 10 m/s). *P*_C_, *P*_CC_, *σ*_VM_, and *τ* produced under different *θ*_H‐W_; cases are summarized in Table 6. The head impact location remarkably influences the brain injuries. The average values of *P*_C_, *P*_CC_, *σ*_VM_, and *τ* are 606.43 ± 100.24, −430.43 ± 50.20, 30.13 ± 3.88, and 16.93 ± 2.27 kPa, respectively.

Then, the FE head model is rotated around the coronal axis to test another set of head-windshield collision angles, the intersection angle between human coronal section, and the tangent plane of the windshield as the impact posture for parametric study. The collision angles are selected based on the realistic situation (Figure 12(e)).  Table 7 shows that the brain injury indices vary greatly with the change in head-windshield collision angles. For instance, *P*_C_, *P*_CC_, *σ*_VM_, and *τ* are relatively high at *α*_H-W_ = 60° and *α*_H-W_ = 100° but with low values in the case of *β*_H-W_ = 60°. The average values of *P*_C_, *P*_CC_, *σ*_VM_, and *τ* are 691.06 ± 311.39, −596.28 ± 325.27, 31.32 ± 4.82, and 17.86 ± 2.89 kPa, respectively. Note that the shape of the brain model is not absolutely symmetrical in all directions. Different contact surfaces of the head are bound to bring change in the values of pressures and stresses.

## 4. Conclusion

In this paper, the multirigid body human and FE vehicle models are coupled to simulate the potential crash accidents. The initial factors on ESS riders' brain injuries subjected to the windshield contact are examined by varying the vehicle impact speed and the ESS moving speed. The results show that the brain injury indices increase with head-windshield impact speed. The ESS moving speed also has an influence on the head-windshield contact. Injury indices are relatively large under high vehicle speed (15 m/s) and ESS moving speed (3 m/s) impact situations. However, no obvious correlations can be found between ESS moving speed and brain injury indices due to the joint action of relative head-windshield impact speed, impact location on the windshield, and head impact area. Series of designed parametric studies proved that the values of brain injury indices have positive correlations with both linear and angular velocities of the head. The results may remove a critical barrier for forensic analysis and accident reconstruction and may be used to guide vehicle/ESS safety designs.

It should be noticed that there are also limitations with this study. Firstly, we found there is no video resource of real-world vehicle ESS impact accident for reconstruction and comparison. Secondly, it saved us loads of hours by using MB pedestrian model and head model instead of whole-body model to analyze the head and brain injuries of ESS rides, but in the meantime, it may result in a slight difference in kinematics [42]. Thirdly, the reference values of stresses or pressures of the FE head model used in this study are not clear even though it does not affect the model and could be used comparing the injuries at different impact conditions. Fourthly, since there is a tiebreak contact interface between the skull and brain of the FE head model which may allow sliding with separation; then, the accuracy of the results may be influenced by this unphysical behavior. Last but not the least, we studied the vehicle speed, ESS speed, head-windshield impact speed, impact location on the windshield, and head impact region effect on brain injuries. There are certainly other influence factors such as vehicle front shape [43], which could be taken into consideration in future studies.

## Figures and Tables

**Table 1:** Simplified force-deflection data of a doublewheel ESS model.

Wheel		Frame		Handlebar	
Deflection (m)	Force (N)	Deflection (m)	Force (N)	Deflection (m)	Force (N)
0	0	0	0	0	0
0.0015	4000	0.0012	1500	0.04	5000
0.002	9000	0.0054	2000	0.07	10,000
		0.0103	3000		
		0.0161	4000		
		0.0293	6500		
		0.0358	6750		
		0.055	6950		

**Table 2:** Simplified force-deflection data of a solowheel ESS model.

Wheel		Pedal		Board	
Deflection (m)	Force (N)	Deflection (m)	Force (N)	Deflection (m)	Force (N)
0	0	0	0	0	0
0.0015	4000	0.0012	1500	0.04	5000
0.002	9000	0.0054	2000	0.07	10,000
		0.0103	3000		
		0.0161	4000		
		0.0293	6500		
		0.0358	6750		
		0.055	6950		

**Table 3:** Parameter settings of polyvinyl butyral laminated glass mode.

Description	Variable	Outer glass	Inner glass	PVB
Mass density	Rho (kg/m^3^)	2500	2500	200
Poisson's ratio for glass	PRG	0.23	0.23	—
Young's modulus for glass	*E*_g_ (GPa)	100	68	—
Yield stress for glass	SYG (MPa)	110	16	—
Plastic hardening modulus for glass	ETG (GPa)	50	60	—
Plastic strain at failure for glass	EFG	0.0004	0.00024	—
Young's modulus for polymer	*E*_p_ (MPa)	—	—	280
Poisson's ratio for polymer	PRP	—	—	0.495
Load curve ID defining effective stress versus effective plastic strain	LCSS	—	—	1360/s

**Table 4:** Collision times of head-windshield contacts at different vehicle impact speed conditions.

Impact condition	Time of head-windshield contact (doublewheel)	Time of head-windshield contact (solowheel)
*V*_C_ = 5 m/s	295 ms	376 ms
*V*_C_ = 7 m/s	224 ms	265 ms
*V*_C_ = 9 m/s	182 ms	210 ms
*V*_C_ = 11 m/s	155 ms	167 ms
*V*_C_ = 13 m/s	136 ms	139 ms
*V*_C_ = 15 m/s	124 ms	129 ms

**Table 5:** Brain injury indices at each impact point of windshield under head-windshield impact speed of 10 m/s.

Impact point	*P*_C_ (kPa)	*P*_CC_ (kPa)	*σ*_VM_ (kPa)	*τ* (kPa)
A1	755	−297	34.7	18.8
A2	767	−558	36.3	19.4
A3	721.7	−407.7	36.5	19.7
A4	772	−355	37.6	20.7
A5	737	−426.5	37.17	20.6
B1	711	−550	33.5	18
B2	691.9	−546.6	36.4	19.5
B3	1066	−428	36.8	19.9
B4	786	−374	37.4	20.6
B5	782	424.6	37.4	20.9
C1	700	−338	34.7	18.6
C2	731	−362	35.3	18.9
C3	723.7	−399	37	20.1
C4	761	−428.8	37.1	20.5
C5	881	−459	37.9	21.1

**Table 6:** Brain injury indices for each head-windshield collision posture (head is rotated about the vertical axis) under head-windshield impact speed of 10 m/s.

*θ*_H‐W_ (rad)	*P*_C_ (kPa)	*P*_CC_ (kPa)	*σ*_VM_ (kPa)	*τ* (kPa)
0	653	−456	33.5	19
*π*/6	579	−462	28.7	15.7
*π*/3	798	−468	34	19.6
*π*/2	621	−385	33.7	18.5
2*π*/3	485	−343	30.3	17.3
5*π*/6	542	−478	24	13.7
*π*	567	−421	26.7	14.7

**Table 7:** Brain injury indices for each head-windshield collision posture (head is rotated about the coronal axis) under head-windshield impact speed of 10 m/s.

Head-windshield impact posture	*P*_C_ (kPa)	*P*_CC_ (kPa)	*σ*_VM_ (kPa)	*τ* (kPa)
*α*_H‐W_ = 0^ο^	653	−456	33.5	19
*α*_H‐W_ = 20^ο^	530	−370	35.3	19.9
*α*_H‐W_ = 40^ο^	683	−677	33.2	19.1
*α*_H‐W_ = 60^ο^	1066	−1031	35.1	20.2
*α*_H‐W_ = 80^ο^	618	−626	38	22
*α*_H‐W_ = 100°	1414	−1292	34	19.7
*β*_H‐W_ = 0^ο^	567	−421	26.7	14.7
*β*_H‐W_ = 20^ο^	460	−330	26.7	15.3
*β*_H‐W_ = 40^ο^	438.6	−326.8	26.9	15.1
*β*_H‐W_ = 60^ο^	481	−433	23.8	13.6
